# Intrinsic Rewards and Employee's Performance With the Mediating Mechanism of Employee's Motivation

**DOI:** 10.3389/fpsyg.2021.563070

**Published:** 2021-07-15

**Authors:** Faiza Manzoor, Longbao Wei, Muhammad Asif

**Affiliations:** ^1^Department of Agricultural Economics and Management, School of Public Affairs, Zhejiang University, Hangzhou, China; ^2^School of Public Affairs, Zijingang Campus, Zhejiang University, Hangzhou, China

**Keywords:** intrinsic rewards, employee motivation, employee performance, small and medium enterprises, Pakistan

## Abstract

The prime goal of this study is to analyze the impact of intrinsic rewards on the performance of an employee. It also focuses on the role of motivation of the employee as an intervening factor. To achieve this objective, data have been collected through the questionnaire method from small and medium enterprises of Pakistan. A total of 400 questionnaires were distributed to the target population, and 300 were received. To test the hypotheses, the confirmatory factor analysis and the structural equation modeling have been used. The main results of the study have shown a positive and significant impact of intrinsic rewards on the performance of the employee. Specifically, the study reveals that the motivation of an employee significantly mediates the association between intrinsic rewards and the performance of the employee. In the light of the findings, implications are outlined.

## Introduction

It is a general presumption that the motivation of an employee plays a pivotal role in amplifying his/her productivity and performance. To attain maximum achievement in the organizations, it is inevitable that the employees must perform optimally. It is a unanimous consensus that workers will accomplish their tasks better when they are highly motivated. Particularly, in developing countries like Pakistan, the personnel are more inclined to perform when they get recognition from the management (Tehseen and Hadi, 2015). The recognition of their achievements may be translated into intrinsic rewards; and through these rewards, the employees may motivate and perform up to their maximum capacity. Earlier literature is evident that there is an affirmative connection between employee motivation and job performance. For instance, Kuvaas et al. (2017) discussed the role of employees, intrinsic and extrinsic motivation, and their performance in the finance trade sector and as store managers, Norway. Their study concluded that intrinsic and extrinsic rewards are considered a principal motivator for the employees. Before this, Grant (2008) explained that motivation results in instant performance and productivity by the employees, and as a result of motivation, employees are self-driven.

Every organization needs financial, physical, and human resources to achieve its targeted goals. It is possible only when motivated employees use their full potential to do the work. Kuvaas and Dysvik (2009) argued that employees who are highly engaged and more willing to do their work take responsibilities as motivated employees. Motivation is not clearly understood nor practiced. Knowledge about human nature is very important for understanding motivation but human nature is not as simple to understand because every human is different from others. Organizations are using different human resource tactics and practices to motivate their employees (Manzoor et al., 2019a). Reward management system and participation of employees in decision-making are frequently used practices by organizations to accomplish their objectives (Güngör, 2011).

The reward management system includes intrinsic rewards and extrinsic rewards like salary, bonuses, recognition, praise, flexible working hours, and social rights (Skaggs et al., 1991). With the help of a reward management system, enterprises can appeal, retain, and motivate employees to attain high performance of the employee (Liu et al., 2008). Gabriel et al. (2016) examined the relationship between effective management of rewards on the performance of employees in the public service sector of Anambra state, Nigeria. They concluded that intrinsic rewards like employee development, recognition, and pay/salary have a significant and positive effect on the performance of employees in the public service of Anambra. They further deduced that the motivation of employees is one of the significant factors for all firms because it enhances the performance of the employee and the performance of the firm.

Based on the above literature, it is evident that intrinsic rewards are one of the main factors that influence the motivation of an employee that has subsequent effects on amplifying the performance of the employee.

Small and medium enterprises (SMEs) are considered as the fundamental tool for economic growth, and they are playing an essential and vital role in the economic and social configuration of the nation (Ahmedova, 2015; Manzoor et al., 2021b). The worldwide perception of small and micro-businesses or firms has reached noteworthy importance in the economic progress of a nation (Kuzilwa, 2005). It is generally claimed that there is no universally accepted definition for SMEs. In fact, it is difficult to adopt a universal definition for SMEs due to differences in firm size, sectors, culture, and the development status of economies in which SMEs operate (Kushnir, 2010). Gibson and Van Der Vaart (2008) proposed a new quantitative formula for defining SMEs that takes into account the revenue of a company and the country-specific economic context in which the SME operates. The definition of SME as defined by the Government of Pakistan is “SMEs are enterprises whose employment size is up to 250, with paid up capital to Rs. 25 million and an annual sales value up to Rs. 250 million” (Perera and Chand, 2015). The report of the United Nations Industrial Development Organization (2002) defines SMEs in terms of the number of employees and classifies these SMEs in developing and developed economies. According to the UNIDO (2002), in developing countries, there are between 5 and 9 employees for small enterprises and between 20 and 99 for medium-sized enterprises (Abor and Quartey, 2010). Pakistan is a developing country that faced a lot of problems including the high unemployment rate, slow growth in the development process, and severe poverty (Manzoor et al., 2019b). As far as the SME sector is concerned, it provides the framework for a developing and inclusive society through employment opportunities. It strengthens the ability among the members of societies to apply their human competencies and develops a strong association with socio-economic development (Van Kleef and Roome, 2007). Unfortunately, in Pakistan, there are some constraints in this sector, such as weak infrastructure, lack of financial resources, low financial allocation and low participation, lack of incentive for staff, and lack of political commitment. In such cases, the retention of workers in the enterprise is very challenging. To address these issues, this study is being conducted. Many empirical studies (Allen and Kilmann, 2001; Ajila and Abiola, 2004; Hafiza et al., 2011) have been conducted on reward system and employee performance. The study by Ajila and Abiola (2004) showed that reward package can influence on employee performance. Based on their findings, they concluded that reward system helps to increase employee performance by enhancing employee skills, knowledge, and abilities in order to achieve organizational objectives. According to the study by Allen and Kilmann (2001), reward practices play a vital role in improving employee performance and to achieve organizational goals. As mentioned earlier, many researchers have identified that employee rewards directly attach to employee performance. In contrast, if an organization fails to reward employees, it will directly affect the performance of the employees. Empirical studies divulge that an efficient reward system can be a good motivator to the employees, but an inefficient reward system can be a de-motivation to the employees in terms of low productivity, internal conflicts, absenteeism, high turnover, lack of commitment and loyalty, lateness and felling grievances. Therefore, an organization needs to develop a strategic reward system for employees in order to retain competent employees, which results in obtaining a sustainable competitive advantage.

Regarding the previous studies, the relationship between intrinsic rewards and employee performance has been considered. However, many researchers have argued that money is not the only motivator for employees to enhance their performance. Jovanovic and Matejevic (2014) argued that once the pay exceeds the subsistence level, intrinsic factors are the stronger motivators, and staff motivation requires intrinsic rewards such as satisfaction at doing a good job and a sense of doing something worthwhile. In contrast, there is an increasing interest and attention on the use of both extrinsic and intrinsic rewards as a performance-related stimulation. Especially in large organizations, they have diverse reward package, and there is a wide literature on their implausible influence in obtaining and retaining highly motivated employees through that. Despite the vast research on the impact of reward in large organizations, a small number of researchers have investigated the case of the SME sector in developing countries like Pakistan. This study contributes by filling the gap in reward literature in the context of the SME sector and identifies whether the SME sector employees in Pakistan value intrinsic rewards the most or not; it tries to explore the attitudes of employees toward the reward policy of their organization. This study is also important as it is relevant for understanding the reward preferences of the SME employees. To conclude, the results of the study may be helpful for exploring the utilization and motivational potential of the reward management in the SME sector of Pakistan. This study attempts (a) to identify the role of intrinsic rewards on job performance and (b) to focus on discovering that employee motivation mediates the relationship between intrinsic rewards and job performance. Therefore, this study is based on an innovative idea that aims to observe the supposed correlation, i.e., to observe the mediating role of employee motivation between the relationship of intrinsic rewards and job performance. The authors ensure that, if organizations recognize the worth of intrinsic reward actions as honestly as possible, they would get best performance from workers. The main objectives of the study are interlinked with each other because conventional motivation theories like the Motivation-Hygiene Theory by Herzberg (Herzberg et al., 1959) unanimously agree that intrinsic rewards have a positive impact on the motivation of the employees, and due to the motivation of the employees, the performance of employees may amplify.

The remainder section of the article consists of the following. The next section explains the point of view of prior researchers who have contributed to analyzing respective variables. Brief existing literature is reviewed followed by research methodology and data collection. Then, empirical results are discussed with the conclusion and future research.

## Literature Review and Hypotheses Development

### Intrinsic Rewards and Performance of Employees

Intrinsic rewards refer to those incentives that have been given to the employees of an organization. An intrinsic reward is an internal reward that employees achieve from completing their tasks or projects successfully. These rewards are mostly psychological and are based on the effort and abilities of a person. Intrinsic rewards elicit a positive emotional reaction and work to motivate employees to continue to improve as well as make lasting behavioral changes when needed (Ryan and Deci, 2020). For example, when someone completes a task successfully, they will often experience a sense of satisfaction and achievement. This intrinsic reward then motivates the employee to continue to complete that task successfully in the future to further experience those positive emotions. Examples of intrinsic rewards in the workplace include pride in your work, feelings of respect from supervisors and/or other employees, personal growth, gaining more trust from managers, doing work that is enjoyable, feelings of accomplishment, learning something new or expanding competence in a particular area, allowing employees to choose which projects they work on, and being part of a team. The prior studies are in favor of the positive consequence of a reward system on the performance of the employees. Devaro et al. (2017) conducted their research in California, and they examined the relationship between training and internal motivation in organizations (profit and non-profit). The study concluded that training has a high frequency in non-profit organizations, and these non-profit organizations have lower base wages as compared with for-profit organizations. According to the study by Tymon Jr et al. (2010), the intrinsic rewards experienced are a critical element in employee retention, satisfaction with the organization, and career success. Stumpf et al. (2013) focused on reducing employee dissatisfaction and withdrawal in major, consultant designed, change programs by increasing intrinsic rewards. The findings of their study showed that intrinsic rewards related positively with satisfaction with the organization and intentions to stay at both time periods, with programs supportive of employee innovation further enhancing employee satisfaction and retention more strongly during the change effort. Furthermore, Mosquera et al. (2020) evaluated the role of satisfaction with intrinsic rewards in the three largest real estate agencies in Portugal. The results of their study indicated that intrinsic rewards have a positive and significant impact on the job satisfaction of the employee. Bassett-Jones and Lloyd (2005) explained that intrinsic motivation and appreciation play a vital role in the satisfaction of employees rather than money and bonuses. Yang (2008) examined the individual performance and outcomes of his study and indicated that we cannot verify individual performance. Even so, he also claimed that if the performance of the employees is observable, then organizations can use direct bonuses or relational contracts to motivate them based on their performance.

Ajila and Abiola (2004) explained that intrinsic rewards have a positive and significant influence on the performance of the employee in an organization. The results further indicate that intrinsic rewards such as career development, responsibility, recognition, and learning opportunities are less influential on the job performance of an employee as compared to extrinsic rewards like pay, bonuses, promotion, and benefits. The employees prefer to get immediate monetary benefits as compared to the recognition of their works. Barber et al. (1992) determined that flexible benefits have a positive association with the performance of employees and satisfaction. Berdud et al. (2016) conducted their study in the healthcare sector of Spain and investigated the connection between incentives and internal motivation of the employees. They have collected the information with the help of interviews. The study concluded that doctors were intrinsically motivated due to two dimensions which included medical practice and pro-social dimension. Based on the above, we hypothesize the following:

**H1:** Intrinsic reward and employee performance have a significant and positive association.

### Employee Motivation and Performance of Employee

The most significant outcome of motivation, arguably, is individual performance. In this regard, intrinsic motivation is posited to garner “the highest degree of effort” (Meyer et al., 2004), since it was related to high energy levels (Ryan and Deci, 2008) and persistence (Vallerand and Blssonnette, 1992). Besides, motivation is completely related to enthusiasm and commitment (Van Den Broeck et al., 2013), thriving (Spreitzer et al., 2005), and well-being (Nix et al., 1999). To energize workers and make them concentrate on their work in an inclusive manner, all these positive-affect states are theorized. Furthermore, to be positively linked to in-role performance in the domains of education, work, and physical (Cerasoli et al., 2014), there has also been evidence of a positive correlation between contextual work performance and creativity (Gagné and Deci, 2005). Furthermore, to boosting performance, motivation energizes a wide range of attitudes, outcomes, thoughts, and emotions. The key benefits of attitudes are the perceptions of autonomy and effectiveness (Cho and Perry, 2012). Yen and Tang (2015) investigated the association between electronic word-of-mouth motivation and hotel attribute performance. Zámečník (2014) suggested that different motivational programs can be organized for the same motivational group of employees. The motivation of the employees and their performance explained that internal and external motivations are important factors for employee performance. Sanyal and Biswas (2014) investigated the attitude of the employees of the software companies in West Bengal (India) toward performance appraisal. They found the best effects of employee motivation toward performance appraisal. Likewise, van der Van Der Kolk et al. (2019) examined the relations among various types of management control, intrinsic and extrinsic motivations, and performance in the public sector. The findings highlighted that intrinsic motivation enhances the performance of the employee. Zlate and Cucui (2015) revealed that performance is closely connected with motivation. His study is intended to present the motivation process within universities as a complex process, which leads to the performance of the personnel only if motivational mechanisms are known and properly applied by University managers. According to the study by Rita et al. (2018), work motivation has a significant effect on employee performance. Kuvaas et al. (2017) investigated the influence of internal and external motivations on employee performance and exposed that both internal and external motivations have a different effect on the job performance of the employee. The findings of the study showed that internal motivation was positively correlated with work performance and has a negative link with turnover intention and burnout. However, extrinsic motivation has a positive relationship with turnover intention and burnout and has a negative correlation with work performance. Kvaløy et al. (2015) concluded that motivation enhances the performance of the employee only after escorted by performance pay. Also, the performance pay reduces if it is not accompanied by motivation. The effect of motivation on organizational performance has been investigated by Osabiya (2015). He concluded that employees should be given the job he has been trained for. Motivated workers perform better than less motivated workers, because motivated workers have some sort of recognition and achievement through motivation. We assume that similar results would be found in the domain of work and thus hypothesize the following:

**H2:** Employee motivation is positively and significantly correlated with employee performance.

### Intrinsic Rewards and Employee Motivation

A reward management system involves the policies, processes, and practices of the organization for rewarding its workers by their skills, commitment, contribution, abilities, and artifice. It is progressed within the reward philosophy, strategies, and policies of the organization and includes agreements in the form of processes, practices, structures, and procedures that will provide applicable styles and standards of compensation, benefits, and other forms of reward (Güngör, 2011). Reward Management System Tool includes both extrinsic and intrinsic rewards, which are also called financial and non-financial rewards. Extrinsic rewards are salary increase, bonus system, prerequisite, etc., whereas intrinsic rewards are; praise and appreciation, title and promotion, responsibility and authority, plague and certificate, education, participation in decisions, design of work, vacation time, social activities, the comfort of working place, feedback, flexible working hours, recognition, social rights, etc. (Yang, 2008).

A basic explanation of motivation is the capability to change behavior. Motivation is a drive that holds one to act because human behavior is directed toward some goal (Kleinginna and Kleinginna, 1981). Social cognitive theory claims that rewards given for the success of challenging performance standards may result in high motivation (Schunk, 1989; Netz and Raviv, 2004). Karami et al. (2013) determined the impact that a reward management system has on employee performance with the mechanism of the mediating role of employee motivation at Isfahan electric company. The results of their study revealed that reward management has a significant positive impact on the performance of the employee, and the motivation of the employee significantly mediated the effect of reward management system on employee performance. Stringer et al. (2011) explored the complex relationships between intrinsic and extrinsic motivations, pay satisfaction, and job satisfaction of the retailer who uses a pay-for-performance plan for front-line employees. The results provide some support for the complementary nature of intrinsic and extrinsic motivations. Intrinsic motivation was positively associated with pay and job satisfactions, whereas extrinsic motivation was negatively associated with job satisfaction and not associated with pay satisfaction. Likewise, Pratheepkanth (2011) assessed the reward system and its impact on employee motivation in commercial Bank of Sri Lanka Plc, in Jaffna District. The aim of his study was to investigate whether rewards and recognition have an impact on employee motivation. The overall findings of the study showed that rewards and recognition have a positive and significant impact on employee motivation. The results also revealed that staff and employees from non-white racial backgrounds experienced lower levels of rewards and motivation. Kuvaas (2006) suggested that employees will take more responsibility when offered developmental opportunities. Motivated employees via rewards are also more engaged and involved with their jobs as compared with employees with low motivation. Accordingly, we propose following hypothesis:

**H3:** Intrinsic reward is positively related to employee motivation.

The earlier literature has concentrated on different dimensions of motivation and its effect on the performance of the employee in the manufacturing sector and large organizations but according to our best knowledge, none of the studies have discussed this relationship in small and medium-sized enterprises. Also, this analysis has different methods compared to previous studies. This study fills the gap in the sphere of knowledge and addresses the role of intrinsic rewards in the performance of employee with the mediating mechanism of employee motivation in the SME sector of Pakistan. Thus, we assume that:

**H4:** Employee motivation will mediate the relationship between intrinsic reward and employee performance.

**Figure 1:**
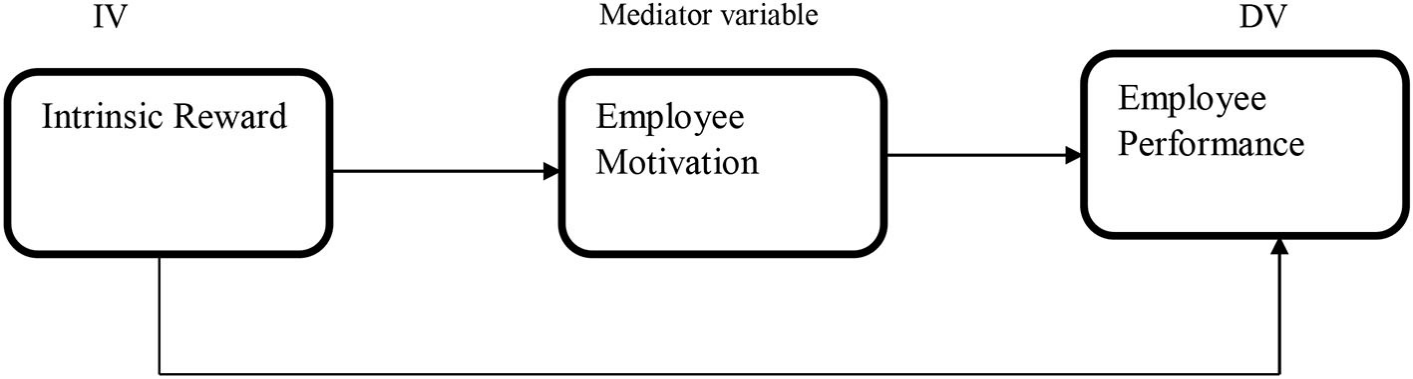
Conceptual framework illustrates the associations examined in this study.

Figure 1 shows the conceptual model of the study, which has been constructed based on a literature review.

## Research Method

### Participants and Procedure

To examine the objective of the study, a sample of employees of SMEs has been collected through the questionnaire. The survey was conducted from early March to late April 2019. These questionnaires were originally developed in both languages English and Urdu (national language) for a better understanding of the local entrepreneurs. Initially, 400 self-administered questionnaires were distributed among the employees of SMEs in Islamabad and Rawalpindi (cities of Pakistan). Study area is chosen due to the density of large numbers of enterprises, and it was convenient for authors to perform the data collection procedure. The SMEs involved in this study were cosmetics, electronics, food and beverage, pharmaceuticals, minerals, and construction. Almost 50 and fewer workers were working in every enterprise.

After the data screening, incomplete questionnaires and unengaged responses were discarded (25% rate); the remaining 300 questionnaires, i.e., 75% of the total participation rate of the respondents, were selected, which were complete in all aspects. The aim of the study and questions were clarified to the participants before giving them a questionnaire, which enabled them to fill the questionnaire easily. The questionnaire consisted of demographic information (control variables such as age, income gender, and education) of the employees and studied variables like an intrinsic reward, employee motivation, and employee performance. The single factor test of Harman (Manzoor et al., 2019c;Sahito et al., 2020) was conducted, and the results showed that the percentage of variance explained by a single factor was far less than 50%, which mean that there is no threat of common method bias.

The total number of respondents was 300, of which 54.3% were males and the remaining were female. The greater part of the respondents (45%) held an undergraduate degree. Almost 26.7% of the age of the respondents was between 29 and 35 years. About 30% of the respondents had 25,000 and 35,000 (PK Rupees) per month income.

### Measures

#### Intrinsic Rewards

Intrinsic rewards have seven items that are taken from the study of Özutku (2012), which were initially developed by Allen and Kilmann (2001). Followers were required to evaluate the intrinsic rewards system using a five-point Likert-type scale ranging from 1 for “strongly disagree” to 5 for “strongly agree.” Sample items included “Regular expressions of appreciation by managers/leaders to employees to acknowledge achievement of quality improvement goals.” and “Quality based promotions wherein promotions are based primarily on the achievement of quality-based goals as opposed to quantity-based goals.”

#### Employee Motivation

The evaluation of employee motivation was performed by six self-report items based on the prior measure (Cameron and Pierce, 1994), and we have retrieved relevant items from the study of Talukder and Saif (2014) and Kuvaas (2006). Items were ranked by a 5-point Likert scale, where 5 = strongly agree and 1 = strongly disagree. Items included “I feel a sense of personal satisfaction when I do this job well.”

#### Employee Performance

Employee performance was measured with the 7-item scale developed by Williams and Anderson (1991), which were previously used in the literature for the assessment of job performance (Arshadi, 2010). Workers were petitioned to rate their level of performance via a 5-point Likert scale, where 5 displays “strongly agree” and 1 displays “strongly disagree.” An example item is “My performance is much better than the same qualified colleagues.”

### Control Variables

This study deals with four control variables, namely, age, gender, income, and education. In this study, we measured the age of the employee through categorical variables (1 = less than 25 years, 2 = 25–29 years, 3 = 30–39 years, 4 = 40–49 years, and 5 = 50 above years), employee gender (1 = male and 2 = female), income (1 = above 55,000, 2 = 45,000–55,000, 3 = 35,000–44,000, 4 = 25,000–34,000, and 5 = below 25,000), and education (1 = no education, 2 = elementary school, 3 = secondary/high school, 4 = bachelor/college, and 5 = master degree/University).

## Results

### Descriptive and Goodness-of-Fit Statistics

SPSS software 22.0 and AMOS 25.0 were used for empirical analysis. Table 1 demonstrates the descriptive statistics, mean, standard deviation, Pearson's correlations, and discriminant validity of all the study variables. The findings depict a positive and significant correlation among all the variables.

**Table 1:** Mean, standard deviation, correlations, and discriminant validity.

***N* = 300**	**Mean**	**SD**	**Correlations**		
			**1**	**2**	**3**
1. Intrinsic reward	3.27	1.30	**(0.904)**		
2. Employee motivation	2.97	1.51	0.50^**^	**(0.844)**	
3. Employee performance	2.66	1.43	0.46^**^	0.73^**^	**(0.861)**

** *Correlations are significant at the p < 0.01*.

*Bold values show discriminant validity and are greater than the squared correlations*.

We have conducted a confirmatory factor analysis (CFA) based on exploratory factor analysis (EFA) and used the maximum likelihood estimate. Table 2 showed the findings of the goodness of fit indices of CFA, where the values of Goodness of Fit Index (GFI) = 0.897, Adjusted Goodness of Fit Index (AGFI) = 0.870, Normed Fit Index (NFI) = 0.945, Relative Fit Index (RFI) = 0.937, Incremental Fit Measures (IFI) = 0.972, Tucker–Lewis Index (TLI) = 0.968, Comparative Fit Index (CFI) = 0.972, Standardized Root Mean Square Residual (SRMR) = 0.064, and Root Mean Square Error of Approximation (RMSEA) = 0.057. All these values surpassed the good fit criteria. According to Bentler and Bonett (1980), the estimates for CFI and NFI should be equal or higher than 0.9 for a good fit, while X^2^/df should be not more than 3. Manzoor et al. (2021a) and Qing et al. (2019) recommended the estimates for NFI and CFI to be above 0.8 for a good fit.

**Table 2:** Goodness-of-fit statistics.

**CFA goodness of fit**	
χ^2^ (chi square)	326.918
Degree of freedom	167
CMIN/DF	1.958
***Absolute fit measures***	
Goodness of Fit Index (GFI)	0.897
Adjusted Goodness of Fit Index (AGFI)	0.870
Normed Fit Index (NFI)	0.945
Relative Fit Index (RFI)	0.937
Incremental Fit Measures (IFI)	0.972
Tucker–Lewis Index (TLI)	0.968
Comparative Fit Index (CFI)	0.972
Standardized Root Mean Square Residual (SRMR)	0.064
Root Mean Square Error of Approximation (RMSEA)	0.057

*CFA, confirmatory factor analysis; Chi square/degree of freedom*.

The convergent validity of the variables was estimated by observing the factor loading, composite reliability (CR), and average variance extracted (AVE) (Table 3). We found that CR ranged from 0.83 to 0.90 for each factor from the results of Table 3. These values are greater than the suggested cut-off point of 0.60 and confirm the existence of inner consistency reliability between each construct (Fornell and Larcker, 1981; Asif et al., 2019a). According to Bagozzi and Yi (1988), the CR ensures the minimum cut-off of 0.60, whereas the AVE crosses the threshold of 0.50 (Fornell and Larcker, 1981). Table 3 shows that the value of alpha is above 0.70 (Cronbach, 1995), which represents the greater internal consistency of the constructs and validity. Hair et al. (2010) suggested that factor loading more than 0.5 is considered significant, and therefore, loadings provide a significant effect for each construct (Han et al., 2019; Asif et al., 2020). Thus, the measures do not have any slight problem with convergent validity.

**Table 3:** Factor loading of indicators and overall reliability of the constructs.

**Items**	**AVE**	**Cronbach's alpha/CR**	**EFA**	**CFA**
**Intrinsic reward**	0.818	0.96/0.969		
Non-monetary form of recognition to acknowledged achievement of quality improved goals such as, merchandise, certificates, and complementary tickets			0.917	0.913
Celebrations to acknowledge achievement of quality improvement goals such as lunches, dinners, and special events			0.876	0.858
Regular expressions of appreciation by managers/leaders to employees to acknowledge achievement of quality improvement goals.			0.901	0.884
360 degrees performance appraisals wherein feedback from co-workers and/or customers is incorporated into performance appraisals.			0.911	0.911
Formal suggestion system available for individuals to make quality improvement suggestions.			0.924	0.920
Use of development-based performance appraisals.			0.900	0.879
Quality based promotions wherein promotions are based primarily on the achievement of quality-based goals as opposed to quantity-based goals.			0.904	0.887
**Employee motivation**	0.713	0.92/0.937		
I feel a sense of personal satisfaction when I do my work well.			0.881	0.882
My view of for myself goes unhappy when I do the work unwell.			0.878	0.880
I feel satisfaction in doing my work well as I can.			0.826	0.813
I feel down when my effort is not up to my standard.			0.843	0.834
I work harder because my subordinates appreciate it.			0.822	0.784
I try to think if ways of doing my work efficiently and effectively.			0.817	0.774
**Employee performance**	0.742	0.94/0.952		
I am aware that the work that I do is important for the organization			0.871	0.859
The work that I perform needs competent personnel, and everyone cannot perform it.			0.884	0.875
The work that I perform is worth doing.			0.842	0.804
I can use my potential completely in my work.			0.846	0.828
My performance is much better than the same qualified colleagues.			0.845	0.843
I mostly fail to complete important responsibilities.			0.881	0.885
I am happy with my performance because it is generally satisfying and better.			0.862	0.870

*AVE, average variance extracted; CR, composite reliability; CFA, confirmatory factor analysis; EFA, exploratory factor analysis*.

To see the discriminant validity, the squared root values of correlations among the constructs are shown in Table 1 where all these values are greater than the inter-related correlations (Asif et al., 2019b). Additionally, the measurement model (shown in Table 4) confirms the construct validity as suggested by Barroso Castro et al. (2008) and Qing et al. (2019).

**Table 4:** Measurement model for explanatory variable (intrinsic reward), dependent variable (employee performance), and mediator variable (employee motivation).

**Indicators**	**Intrinsic reward**			**Employee motivation**			**Employee performance**		
	**Standardized regression weights**	***t***	***R*^**2**^**	**Standardized regression weights**	***t***	**R^**2**^**	**Standardized regression weights**	***t***	***R*^**2**^**
IR1	0.913	Fixed	0.834						
IR2	0.858	22.993	0.736						
IR3	0.884	24.828	0.781						
IR4	0.911	27.099	0.830						
IR5	0.920	27.321	0.846						
IR6	0.879	24.511	0.773						
IR7	0.887	25.051	0.787						
EM1				0.882	Fixed	0.778			
EM2				0.880	21.422	0.774			
EM3				0.813	18.402	0.661			
EM4				0.834	19.294	0.696			
EM5				0.784	17.235	0.615			
EM6				0.774	16.894	0.599			
EP1							0.859	Fixed	0.738
EP2							0.875	20.484	0.766
EP3							0.804	18.105	0.646
EP4							0.828	19.09	0.686
EP5							0.843	19.765	0.711
EP6							0.885	21.799	0.783
EP7							0.870	21.045	0.757

*IR, intrinsic reward; EM, employee motivation; EP, employee performance*.

### Hypotheses Testing

To test the hypotheses of the study, we employed structural equation modeling (SEM) using the maximum likelihood estimation in IBM-AMOS software.

Table 5 shows that intrinsic reward and employee performance have a significant and positive association. As evident in Table 5, we found support for Hypothesis 1 (Standardized β = 0.46, *t* = 9.17, and *p* < 0.01). Hence, intrinsic reward has a positive and significant association with employee motivation (Standardized β = 0.50, *t* = 10.13, and *p* < 0.01); additionally, employee motivation and employee performance have a positive and significant correlation (Standardized β = 0.73, *t* = 18.69, and *p* < 0.01). Therefore, the findings of regression fully support Hypothesis 2 and 3.

**Table 5:** Regression coefficients for a direct relationship of variables for testing hypotheses 1–3.

**Path**	**Standardized B**	**Std. Error**	**T**	**Significance**
Intrinsic reward → Employee performance	0.46	0.056	9.17	0.000(^**^)
Intrinsic reward → Employee motivation	0.50	0.058	10.13	0.000(^**^)
Employee motivation → Employee performance	0.73	0.037	18.69	0.000(^**^)

** *Significant at p < 0.01*.

For testing Hypothesis 4 which is about the mediation effect of employee motivation between intrinsic reward and employee performance, we applied two methods suggested by Baron and Kenny (1986) and James and Brett (1984). The study of Baron and Kenny (1986) was concerned with regression weights and correlation of studied variable, and for full mediation support, four criteria should be met. First, the predictor variable (intrinsic reward) should have a significant relationship with a mediator (employee motivation). Second, the intrinsic reward should have a significant relationship with the predicted variable (job performance). Third, the mediator variable must be significantly correlated with the predicted variable. Lastly, in the regression equation, the direct association among explanatory variables and predicted variables must be insignificant in the existence of a mediator variable.

However, the mediation for the existing study is verified with the help of James and Brett (1984). They recommended adopting confirmatory approaches like SEM to test mediation.

With the recommendation of Wang et al. (2005), we prepared two nested models and compared them as presented in Table 6. Model A is a hypothesized one that has a direct path from intrinsic reward (explanatory variable) to employee performance (outcome variable) and also integrated an indirect path from intrinsic reward to a dependent variable through employee motivation (mediator variable). Further, this model is compared by another model. Model B included an indirect path from the explanatory variable to the dependent variable. Table 6 showed that χ^2^ difference is insignificant while comparing the hypothesized model A. This shows that Model A is the best-fitted model and confirmation of mediation.

**Table 6:** Comparison of the structural equation model.

**Model and structure**	**χ^2^**	**df**	**χ^2^/df**	**Δ χ^2^/df**	**Δdf**	**TLI**	**CFI**	**RMSEA**
A. Hypothesized model	330.27	168	1.966	–	–	0.968	0.972	0.057
B. IR-EM-EP	326.91	167	1.958	0.008	1	0.967	0.970	0.059

*IR, intrinsic reward; EM, employee motivation; EP, employee performance; RMSEA, Root Mean Square Error of Approximation; TLI, Tucker–Lewis Index; CFI, Comparative Fit Index*.

**Figure 2:**
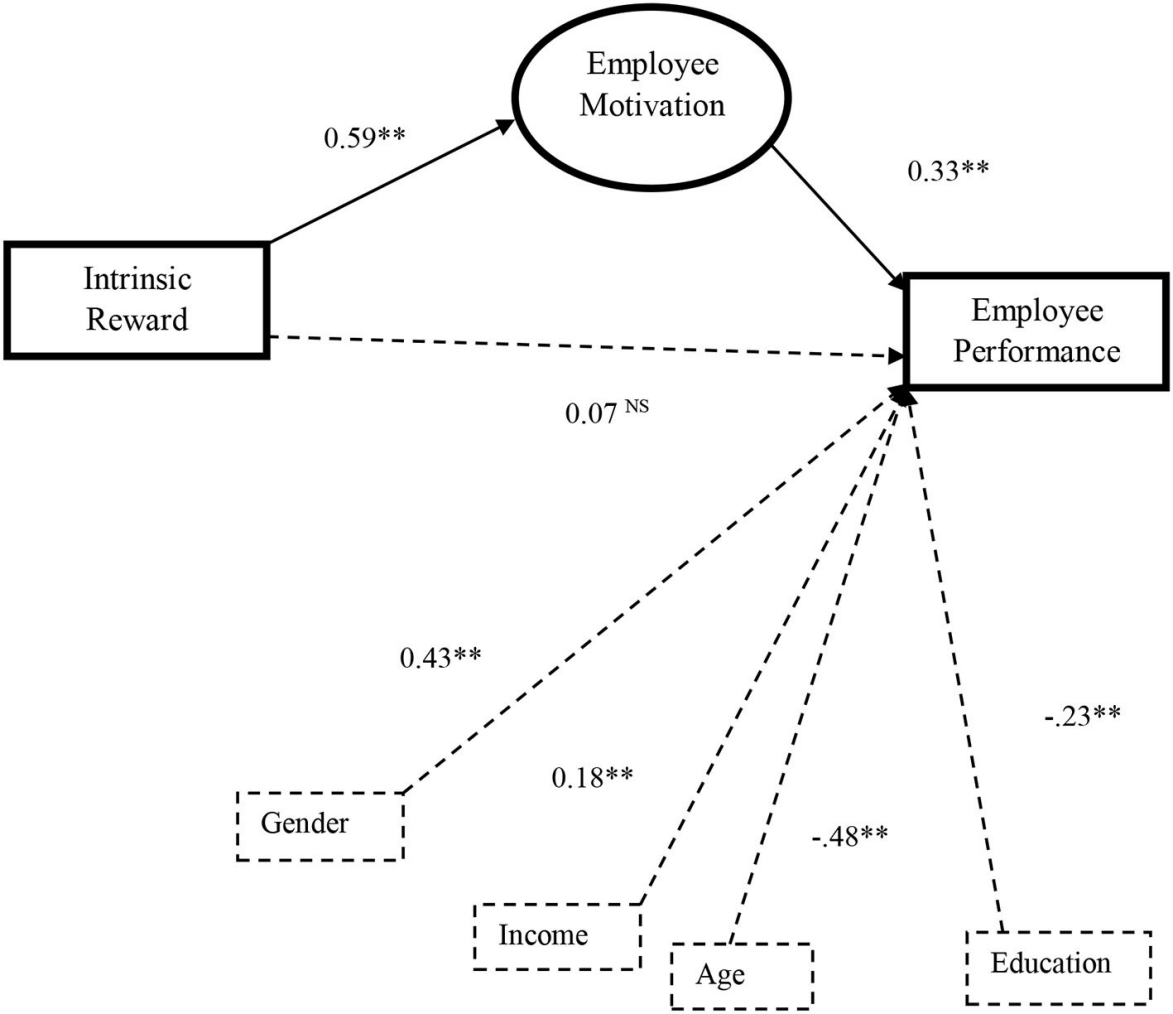
Structural equation modeling mediation effects. Significant at ** *p* < 0.01; *** *p* < 0.001; NS, non-significant.

Furthermore, Figure 2 shows the SEM results where a path from intrinsic reward to employee motivation is significant (β = 0.59; *p* <0.01), whereas the path from employee motivation to job performance also shows a significant and positive association (β = 0.33; *p* < 0.05). From Figure 2, it is evident that the direct path from intrinsic reward to employee performance (β = 0.07, *p* > 0.05) is insignificant and approves full mediation. In line with the above evidence, we performed bias-corrected bootstrapping and percentile bootstrapping at a 95% confidence interval with a 2,000 bootstrap sample (Arnold et al., 2015) to assess complete or partial mediation. As recommended by Preacher and Hayes (2008), we measured the confidence of the interval of the lower and upper bounds to assess the importance of indirect effects. As shown in Table 7, we found that the indirect effects of employee motivation on the intrinsic reward and employee performance (estimate = 0.191, *p* < 0.01) are significant. The direct relationship between intrinsic reward and employee performance (estimate = 0.067, *p* = 0.100) is not significant and supported Hypothesis 4 with complete mediation.

**Table 7:** Results of bootstrapping for standardized direct, indirect, and total effects of the model.

	**Coeff**.	**Std. E**	**Bootstrapping**				
			**Bias-corrected percentile 95%**		**Percentile method 95%**		**Sig**.
			**LLCI**	**ULCI**	**LLCI**	**ULCI**	
**Standardized direct effects**							
IR → EP	0.067	0.040	−0.004	0.154	−0.013	0.148	0.10
EM → EP	0.379	0.107	0.185	0.589	0.163	0.552	^***^
IR → EM	0.506	0.053	0.394	0.601	0.399	0.607	^***^
**Standardized indirect effects**							
IR → EM → EP	0.192	0.057	0.097	0.317	0.080	0.290	^***^
**Standardized total effects**							
IR → EP	0.259	0.072	0.135	0.406	0.108	0.386	^***^
EM → EP	0.379	0.107	0.185	0.589	0.163	0.552	^***^
IR → EM	0.506	0.053	0.394	0.601	0.399	0.607	^***^

*IR, intrinsic reward; EM, employee motivation; EP, employee performance; LLCI, lower level of confidence interval; ULCI, upper level of confidence interval. Sig:*

** *p < 0.05*,

*** *p ≤ 0.01*.

## Discussion

The key aim of this study was to investigate the association between intrinsic reward and employee performance in the presence of employee motivation as a mediator among SME employees. This study has been conducted in the SME sector of Pakistan, and very rare empirical studies have been scrutinized the reward system and its effects on employee performance. This study fills this gap by examining the association between intrinsic reward and employee performance in the context of the SME sector in Pakistan.

In this study, results revealed that the relationship between the intrinsic reward (independent variable) and employee performance is positive and significant. These findings were confirmed by previous studies of Pierce et al. (2003), Cerasoli et al. (2014), and Ajila and Abiola (2004). Furthermore, the outcomes of the existing study revealed that intrinsic reward has a significant and affirmative correlation with employee motivation. These findings allied with the past studies of Cho and Perry (2012), Kuvaas et al. (2017), and Fisher (1978). Also, employee motivation (mediator variable) and outcome variable employee performance also have a significant and positive association, and these findings are consistent with the previous studies of Mak and Sockel (2001), Bedarkar and Pandita (2014), and Khan et al. (2017). The results supported the hypotheses that there is a positive association between intrinsic reward, employee motivation, and employee performance.

Motivation in the workplace has been traditionally understood in terms of extrinsic rewards, be in the form of pay, benefits, bonuses, awards, or career advancement (Rebitzer and Taylor, 2011). However, intrinsic rewards play an important role in a workplace motivational strategy, which makes employees more motivated to work. Many people respond well to tangible intensive rewards, such as a monetary bonus. However, once the reward is depleted, the motivation may also dwindle, so a strong strategy uses both intrinsic and extrinsic rewards to keep workers motivated throughout their tenure (Vallerand and Blssonnette, 1992). The results of our study reveal that intrinsic rewards are vital to motivational success because they offer long-term, non-tangible benefits that are usually not very costly to achieve and can be repeated over and over again successfully.

The mediating effect of employee motivation on the relationship between intrinsic reward and employee performance is our main finding. The result demonstrated that employee motivation has a positive mediating effect in the association between intrinsic reward and employee performance. These results are in line with a previous study of Güngör (2011); he has conducted his study on global banks in Istanbul. This suggests that employees with the best reward system and motivation process can be more satisfied and will exhibit a higher level of job satisfaction. Consequently, their performance will be improved.

This study significantly contributes to the existing literature on intrinsic reward and job performance by investigating the unexplored side of intrinsic reward—the performance of the employee in different ways. First, the previous study showed that a reward management system can significantly influence a workforce and can be motivated to improve their efforts and performance (Rai et al., 2018), and therefore, different rewards have been employed to measure performance such as monetary reward (Aguinis et al., 2013), remuneration (Calvin, 2017), and enough pay and bonus (Pouliakas, 2010); hence, we indicated intrinsic reward for measuring employee performance. Second, only a few studies discovered the relationship between intrinsic reward and employee performance and used some mediators such as organizational commitment (Taba, 2018) and reward system (Riasat et al., 2016), but no study has employed employee motivation as mediators. Third, very limited research has been performed to examine intrinsic reward—the performance of the employee in the Pakistan context by using mediating mechanisms, but no study has explored that in SMEs. Therefore, this is the first study to examine the effects of intrinsic reward on the performance of the employee through employee motivation in Pakistani SME sector.

## Conclusion

This study was conducted to analyze the impact of intrinsic rewards on the performance of the employee with the mediating mechanism of employee motivation in the SME sector of Rawalpindi and Islamabad, Pakistan. The major results of the study disclose that intrinsic rewards have a significant positive impact on the motivation of the employee and employee performance. Another important result is that the motivation of the employee plays a positive and significant mediating role in the association of intrinsic rewards and performance of the employee. It is a general perception that when employees are motivated, they perform better. It means that if organizations have a good reward management system, the motivation of their employees will be high and the performance of their employees will amplify with greater magnitude. In the absence of a good reward management system, their employees will be demotivated, and the performance of their employees will also be declined. The SME sector should develop a sound rewards management system for employees to boost their morale and motivation to get better results. The study is particularly helpful for this sector in understanding the importance of intrinsic rewards and motivation. It is also useful to understand the problems which organizations may face if they do not have a good reward management system.

### Theoretical and Practical Implications

The findings of this study hold important implications both theoretically and practically. The outcomes of this research propose some essential theoretical implications for reward management system literature. First, by testing the mediating role of employee motivation in the relationship between intrinsic reward and employee performance, this study contributes to a superior understanding of the causal mechanism through which intrinsic reward relates to the job performance of the employees. The outcomes of the recent study identify that organizations improve employee motivation by exhibiting intrinsic rewards, which ultimately lead to enhance employee performance. The findings of this study support the current evidence indicating that employee motivation is a significant motivational source that encourages workers to be extra dedicated and satisfied with their job (Grant, 2008; Sledge et al., 2008). Besides, analyzing the motivation of employees as a mediator helps us better understand how and why intrinsic incentives can improve the behavior of employees at work. Second, this study has been conducted in a developing nation “Pakistan,” which is an Asian country, and has a rare study in this area. To date, very few studies have explored the reward management system and its effect on employee performance in the Pakistani SME sector. Interestingly, the study outcomes indicate that intrinsic rewards can be beneficial and effective in enterprises and organizations in the country.

The practical implication of this study is that first, our study confirmed that intrinsic reward is effective in improving employee performance and also suggests that the reward system is very important in the organizations to encourage their employees. Second, as this study illustrates that intrinsic reward has an indirect effect on employee performance in the presence of employee motivation, it is suggested that organizations should inaugurate such conditions through which they can improve the performance of the employees. Organizations should do whatever they can to increase the motivation of the employees. Third, as the intrinsic reward has a positive influence on job performance, firms need to promote a reward management system and motivation. For instance, organizations can promote a reward management system (financial reward and non-financial reward), and this would motivate employees to achieve their goals and promote optimal fulfillment in work.

Another possible way to encouraging employees is to set goals and achieving them provides an intrinsic reward (intangible award, i.e., appreciation, promotion, and authority). A firm should require all workers to set targets for personal development at work, education, and the completion of projects. Provide training to workers on how to fixed measurable objectives and encourage them to set a variety of short- and long-term goals. Give employees input into company goals as well to make them feel like they are working toward a greater cause. As employees meet goals and set new goals, they will receive intrinsic rewards and increase their motivation.

### Study Limitations and Future Directions

It is very essential to view some limitations of this study which can lead to future research. First, the cross-sectional study design was applied for data collection; future studies should use a longitudinal study design to this study model to avoid the ambiguity of a causal relationship. We have studied the relevant source of intrinsic reward and employee motivation in the domain of work, and we used a measure of intrinsic reward that exclusively focuses on intangible incentives. As there are several other sources of tangible reward and extrinsic motivation in most work settings, including handsome salary, bonuses, deadlines, evaluations, and surveillance, future work could develop new and broader measures. For future research, other factors of employee performance like employee efficiency, job achievement, and job fulfillment should be considered while seeing the impact of rewards. It is also recommended for future researchers to check the effects of intrinsic and extrinsic rewards on the performance of the employee in other sectors like education, health, etc. By taking a larger sample of the employees, their comprehensive performance may be judged in other developing countries. Besides, this study was performed in the context of one developing nation, Pakistan. Future studies should be carried out to boost the generalizability of the findings by testing this model in other developing countries. Moreover, it would be interesting in the future to explore the moderating role of motivation of the employee between the reward management system and job performance.

## Data Availability Statement

The raw data supporting the conclusions of this article will be made available by the authors, without undue reservation.

## Ethics Statement

The studies involving human participants were reviewed and approved by the research Ethical Committee of Zhejiang University, Hangzhou, China. The participants provided their written informed consent to participate in this study.

## Author Contributions

FM initiated the basic idea and wrote the main part of the manuscript and built the article structure. LW reviewed and improved the manuscript. MA contributed to the methodology of this study. All authors contributed to the article and approved the submitted version.

## Conflict of Interest

The authors declare that the research was conducted in the absence of any commercial or financial relationships that could be construed as a potential conflict of interest.
